# 100 Years of BCG Immunization: Past, Present, and Future

**DOI:** 10.3390/vaccines10101743

**Published:** 2022-10-18

**Authors:** Aldo Tagliabue, Diana Boraschi, Luciana C. C. Leite, Stefan H. E. Kaufmann

**Affiliations:** 1 Shenzhen Institute of Advanced Technology (SIAT), Chinese Academy of Sciences, Shenzhen 518055, China; 2 Institute of Biomedical Technologies (ITB), National Research Council (CNR), 20054 Segrate, Italy; 3 Institute of Biochemistry and Cell Biology (IBBC), CNR, 80131 Napoli, Italy; 4 Stazione Zoologica Anton Dohrn, 80121 Napoli, Italy; 5 Instituto Butantan, São Paulo 05503900, Brazil; 6 Max Planck Institute for Infection Biology, 10117 Berlin, Germany; 7 Max Planck Institute for Multidisciplinary Sciences, 37077 Göttingen, Germany; 8 Hagler Institute for Advanced Study, Texas A&M University, College Station, TX 77843-3572, USA

The 100th anniversary of the introduction of Bacille–Calmette–Guérin (BCG) as a tuberculosis (TB) vaccine is an occasion warranting further investigation of the early attempts which culminated in the introduction of BCG as a TB vaccine, as well as of subsequent recognition of failures, new findings that broaden its applications, outstanding questions, and approaches towards the development of novel vaccine candidates [1,2].

It all started with the announcement by Robert Koch in 1890 that he had developed a remedy to prevent and treat TB, which, in less than two years, turned out to be a disastrous failure [3]. Subsequently, various approaches were attempted with no success (low doses of *Mycobacterium tuberculosis* (Mtb), short-term attenuation of *M. tuberculosis* by biophysical methods, and inactivated whole cell and split vaccines based on *M. tuberculosis* or other mycobacteria). Ultimately, Albert Calmette and Camille Guérin combined the strategy of Edward Jenner, i.e., to use a pathogen of different host specificity, and that of Louis Pasteur, i.e., to employ attenuated pathogens, using long-term in vitro passage of *Mycobacterium bovis*, the agent of cattle TB, for attenuation. Eventually, Bacille–Calmette–Guérin (BCG), as it was termed, was shown to be successful. Originally, the vaccine had been given orally, but was soon found to be more efficacious with intradermal inoculation. The success story of BCG was profoundly affected by the so-called Lübeck disaster. In 1930, 251 neonates had been vaccinated with BCG preparations that were accidentally contaminated with fully virulent *M. tuberculosis*, ultimately leading to the death of 75 babies [4,5]. Over the years, however, BCG stood the test of time, and it remains the only licensed vaccine against TB today.

On the other hand, it is increasingly being recognized that BCG can no longer be considered as one vaccine. In fact, due to varying culture conditions, the different BCG strains currently used have given rise, over the years, to many substrains. These substrains have shown a variety of different genetic characteristics and immunological properties. Furthermore, several different culture media formulations are currently used worldwide for production of the different strains, a circumstance that can substantially influence the expression of BCG surface molecules, thereby modifying their interaction with host cells [6].

Yet, it has become clear that BCG primarily protects against extrapulmonary TB in infants and is of variable efficacy against pulmonary TB in all age groups. Hence, improved vaccines to protect infants, adolescents, adults, and the elderly against the most prevalent disease form, pulmonary TB, are urgently needed. In addition, future TB vaccines should be safer than BCG.

Among the various strategies currently investigated for improving anti-TB efficacy, there exist four promising approaches. These include subunit vaccines composed of one or few antigens and whole cell vaccines comprising a multitude of antigens. The four strategies are:

- Mycobacterial proteins combined with potent adjuvant formulations (as presented in refs. [7,8]);
- Viral vectors (such as MVA and Adenovirus) expressing mycobacterial antigens [9,10];
- Inactivated whole cell vaccines, in general using related mycobacteria [11];
- Viable mycobacterial vaccines comprising either recombinant BCG (rBCG) overexpressing different mycobacterial antigens or proteins that can modulate the immune response (see for instance ref. [12]), or attenuated Mtb (such as MTBVAC [13]).

An rBCG strain expressing a detoxified *E. coli* toxin showed improved efficacy against TB in animal models, but also a better capacity to induce a non-specific innate/inflammatory response in human macrophages [14]. Further advanced is the rBCG-expressing Listeriolysin O (VPM1002) to perturbate phagosomal membranes, which has already successfully passed safety and immunogenicity trials [15,16].

This reawakening of TB vaccine development has also led to the reevaluation of BCG itself. It was revealed that in non-human primates BCG could induce sterilizing immunity after intravenous inoculation [17], although splenomegaly was observed as an adverse event. In human trials, compelling evidence was obtained suggesting that revaccination of adults with BCG can prevent stable infection by almost 50% [18].

Today, a dozen vaccine candidates have entered clinical testing based on these four approaches. The only novel vaccine candidate that has shown preliminary efficacy data in a phase IIb trial is the subunit vaccine M72:AS01_E_ (protein + adjuvant), which demonstrated around 50% prevention of disease after booster vaccination of individuals with latent TB infection [4]. The most advanced vaccine candidate is VPM1002 (an rBCG with improved immunogenicity and safety) [15,16], which is currently being tested in three phase III clinical trials for prevention of infection, prevention of disease, and prevention of recurrence in different study populations (for review see ref. [2]).

Additional new attributes in the use of BCG include its non-specific innate/inflammatory effects, encompassing the generation of an innate memory able to protect against non-related infections [19,20,21]. This property has herein been demonstrated to extend to rBCG strains. A recombinant strain expressing a detoxified pertussis toxin antigen displayed an enhanced capacity to induce innate memory, both in vitro, in macrophages, and in vivo, in response to unrelated infectious challenges [22]. Beyond infections, BCG is also being used successfully for therapy of non-muscle-invasive bladder cancer [23]. BCG, however, is not accepted by all bladder cancer patients and VPM1002 has recently been shown to significantly prolong survival time in such patients [24].

Recombinant BCG has also been used as a strategy to develop vaccines against non-related pathogens, joining its capacity to non-specifically enhance immune responses with the stimulation of an antigen-specific response. Thus, the expression of protective antigens in BCG has shown the induction of potent immune stimulation in response to the heterologous pathogens (see for instance ref. [25]). The use of complemented auxotrophic BCG strains improves vaccine stability, and there are several examples of its applicability both for new anti-TB vaccines and as live vectors for vaccines against different diseases (reviewed in ref. [26]).

What is the future for BCG? Its use as a vaccine can be effectively complemented by a thorough exploitation of its immunostimulatory capacities, which can be directed to targeted adjuvanticity, to the amplification of specific secondary responses, and to broaden the spectrum of vaccine efficacy, as well as to the stimulation of a localized inflammatory/cytocidal innate immune reaction against tumors (reviewed in ref. [27]).

With the celebration of the first 100-year anniversary of the most employed vaccine in the world prior to COVID-19, there have been a few special topic issues and several articles published in the last year in a variety of journals. Scientific progress in vaccine research and development has accelerated with an enormous speed, but what we have learned from BCG discovery more than 100 years ago will remain the basis of vaccine research and development forever. Confirming Isaac Newton’s phrase, “Standing on the shoulders of giants”, we can assuredly say: “Today’s research and development stands on the shoulders of the giants who solved the key issues more than 100 years ago”.
